# USABILITY TESTING OF THE PACE APP TO SUPPORT FAMILY CAREGIVERS IN MANAGING PAIN FOR PEOPLE WITH DEMENTIA

**DOI:** 10.1093/geroni/igad104.2763

**Published:** 2023-12-21

**Authors:** Nai-Ching Chi, Angela Shanahan, Kristy Nguyen, Ibrahim Demir, Ying-Kai Fu, Keela Herr

**Affiliations:** University of Iowa, Iowa City, Iowa, United States; University of Iowa, Iowa City, Iowa, United States; University of Iowa College of Nursing, Iowa City, Iowa, United States; University of Iowa, Iowa City, Iowa, United States; University of Iowa, Iowa City, Iowa, United States; University of Iowa, Iowa City, Iowa, United States

## Abstract

Pain is a common symptom in older adults with Alzheimer’s Disease and Related Dementias (ADRD). It is difficult to assess and is often undertreated in people with ADRD. The PACE-app (Pain Control Enhancement application, was developed to enhance self-efficacy of family caregivers when controlling the pain of their care partner. The objective of this study was to test the usability of the PACE-app with family caregivers and clinicians. We evaluated the usability of the PACE-app. Participants (17 family caregivers, 6 clinicians) took part in a mixed-methods study that included a one-time recorded zoom meeting with standardized semi-structured guided review, interview, and a survey specific to their role. The Post-Study System Usability Questionnaire (PSSUQ) was used to measure usability. The overall PSSUQ score we found was 1.9. The range of scores can be between 1 and 7 with the lower numbers meaning favorable usability. The qualitative interviews showed a favorable user experience with 75% of all participants feeling the overall design was effective; 48% felt the tailored pain management strategies were useful; 24% suggested improving the app by having direct coaching information for the family caregiver. This study demonstrated favorable usability with those who tested the PACE-app. Suggestions for improvement were given and using the interview results in conjunction with the PSSUQ scores, we are able to make changes to the app interface to impact the overall design to enhance user experience.

